# Polyploidization increases meiotic recombination frequency in *Arabidopsis*: a close look at statistical modeling and data analysis

**DOI:** 10.1186/1741-7007-10-30

**Published:** 2012-04-18

**Authors:** Lin Wang, Zewei Luo

**Affiliations:** 1 Laboratory of Population & Quantitative Genetics, Institute of Biostatistics, Fudan University, Shanghai 200433, China; 2 School of Biosciences, University of Birmingham, Birmingham B15 2TT, UK

## Abstract

This paper is a response to Pecinka A, Fang W, Rehmsmeier M, Levy AA, Mittelsten Scheid, O: **Polyploidization increases meiotic recombination frequency in *Arabidopsis***. *BMC Biology *2011, **9**:24.

See research article at http://www.biomedcentral.com/1741-7007/9/24

## Background

Many species, particularly flowering plants, have usually experienced a state of polyploidy in their evolutionary history. Meiotic recombination creates novel configurations of genetic variants maintained in the genome of a species for facilitating natural and artificial selection. Thus, to understand how a diploid species differs in frequency of meiotic recombination from its polyploid ancestor has a significant impact in both evolutionary biology and plant and animal genetic breeding. It has been well established that the evolution of polyploid genomes is an extremely dynamic process compared to that of diploids, characterized by extensive genetic and epigenetic changes occurring in the nuclear genome following polyploidization [1-3]. Little is known about the mechanism underpinning the genetic changes. To address this fundamental question, Pecinka and colleagues described in a recently published paper by *BMC Biology *a direct comparison in frequency of meiotic recombination of the diploid and tetraploid genomes of *Arabidopsis *[4]. One of the most striking methodological challenges to the study is to properly evaluate the recombination parameter in populations of the species at different levels of polyploidy, particularly in autopolyploids. In fact, linkage analysis with autotetraploids has been a historical problem that can be traced back to the pioneering works of the prominent mathematical geneticists [5-7].

There are at least two major challenges to the tetrasomic linkage analysis. Firstly, double reduction, the most distinct characteristic of polysomic inheritance, allows sister chromatids to enter into the same gamete during meiosis and thus cause systematic distortion in allele segregation [8]. Secondly, multiplex allele segregation makes it almost impossible to infer the underlying genotype directly from PRC-based phenotype data even for co-dominant markers such as a single nucleotide polymorphism or simple sequence repeats [9]. To avoid these difficulties, these authors firstly developed a seed-based assay by creating transformants with green and red fluorescent markers expressed under a seed-specific promoter in *Arabidopsis thaliana *[10,11]. In parallel, they created diploids, allotetraploids and autotetraploids which carry these fluorescent markers. After carrying out a series of backcross and selection breeding, they were able to create the diploid and tetraploid lines which bear only a single copy of the marker alleles linked on the *Arabidopsis *chromosome III. These lines were used to create the segregation populations from which marker phenotype data were collected and used for estimation of recombination frequency between the markers [4].

The method these authors implemented for modeling and analyzing the marker data from diploid and tetraploid (allo- and autotetraploid) populations needs to be formulated on the basis of the disomic and tetrasomic inheritance models. This paper presents statistically appropriate and mathematically rigorous methods for modeling and re-analyzing the datasets.

### Notation, model and analysis

We consider segregation of alleles at the two fluorescent marker loci on the *Arabidopsis *chromosome III in a F_2 _family from crossing two parental lines at the marker loci. Following notations of Pecinka *et al. *[4], parental genotypes at the markers can be denoted by GR/GR and BC/BC for diploid, GR/BC/DE/DE and BC/BC/DE/DE for allotetraploid, and GR/BC/BC/BC and BC/BC/BC/BC for autotetraploid. The parental lines were crossed to generate offspring populations of diploids, allotetraploids and autotetraploids accordingly. Regardless of polyploidy, the offspring populations from mating these parents can be grouped into four phenotypes: yellow (carrying both red and green marker alleles), green (green allele only), red (red allele only) and grey (none of the marker alleles). The number of individuals for each of the four phenotype classes is denoted by *n*_1_,*n*_2_,*n*_3 _and *n*_4 _respectively. Let *r *be recombination frequency between the two markers and α be the coefficient of double reduction at the green marker, which is nearer to the centromere than the red marker locus. The probability of observing each of the four phenotypes in the diploids and allotetraploids depends on only one parameter, *r *(*f_i_*(*r*), *i *= 1,...,4), but characterization of the phenotypic distribution in the autotetraploids needs the two parameters, *r *and α (*f_i_*(*α*,*r*), *i *= 1,...,4).

The logarithm of the model parameter(s) given the observations *n*_1_,*n*_2_,*n*_3 _and *n*_4 _can be written as:

$$L\left(r|n_{1},n_{2},n_{3},n_{4}\right)\propto \overset{4}{\underset{i=1}{\sum }}n_{i}\log\left[f_{i}\left(r\right)\right]$$

for the diploid and allotetraploid populations or:

$$L\left(\alpha ,r|n_{1},n_{2},n_{3},n_{4}\right)\propto \overset{4}{\underset{i=1}{\sum }}n_{i}\log\left[f_{i}\left(\alpha ,r\right)\right]$$

for the autotetraploid population where *f_i_*(*r*) (*i *= 1,...,4) can be worked out following the principle of two-locus disomic linkage analysis and listed in Table 1. It needs to be pointed out that the phenotypic distribution is common between the diploid and allotetraploid populations. This is because homoeologous pairing was completely excluded in meiosis of the synthesized allotetraploids of *Arabidopsis *[10,11], thus the allotetraploids show strict disomic inheritance. However, calculation of phenotypic distribution in the autotetraploid segregation population must follow the principle of tetrasomic linkage analysis as is detailed in [11]. In the present context, the phenotypic distribution can be worked out as:

**Table 1:** Distribution of seed phenotype and the underlying genotype at the two fluorescence markers in F_2 _diploid and autotetraploid populations and estimates of the model parameters

	Diploids/Allotetraploid				Autotetraploids		
	
Phenotype	Genotype^a^	Frequency (*f_i_*(*r*))	Obs (*n_i_*)	Obs (*n_i_*)	Genotype^b^	Frequency^c ^	Obs (*n_i_*)
Yellow	G_R_	3(1-*r*)^2^/4+*r*(1-*r*)+*r*^2^/2	2,805	1,484	G^(*i*)^B^(4-*i*)^R^(*j*)^C^(4- *j*)^	*f*_1_(*α*,*r*)	12,707
Green	G_CC	*r*(2-*r*)/4	322	275	G^(*i*)^B^(4-*i*)^C^(4)^	*f*_2_(*α*,*r*)	1,868
Red	BBR_	*r*(2-*r*)/4	333	298	B^(4)^R^(*i*)^C^(4- *i*)^	*f*_3_(*α*,*r*)	2,216
Grey	BBCC	(1-*r*)^2^/4	791	320	B^(4)^C^(4)^	*f*^4^(*α*,*r*)	3,098

Estimates	$\hat{r}\pm s.d.$	Log-likelihood	$\hat{r}\pm s.d.$	Log-likelihood	$\hat{\alpha }\pm s.d.$	$\hat{r}\pm s.d.$	Log-likelihood
	
Present study	0.1643 ± 0.0062	-4,177.79	0.2770 ± 0.0110	-2,553.43	0.0676 ± 0.0121	0.3048 ± 0.0051	-20815.3
Pecinka *et al.*	0.154 ± 0.009	-4,179.20	0.241 ± 0.018	-2,559.21	-	0.205 ± 0.011	-21010.0

^a^Stands for any other alleles; ^b^G^(*i*)^B^(4-*i*)^R^(*j*)^C^(4- *i*) ^is a tetraploid genotype containing *i *'G' alleles, 4-*i *'B' alleles, *j *'R' alleles and 4 - *j *'C' alleles; ^c^forms of *f_i_*(*α*,*r*) (*i *= 1, 2, 3, 4) are presented in context. s.d.: standard deviation.

$$f_{1}\left(\alpha ,r\right)=g_{1}\left(\alpha ,r\right)\left[2-g_{1}\left(\alpha ,r\right)\right]+2g_{2}\left(\alpha ,r\right)g_{3}\left(\alpha ,r\right)$$

$$f_{2}\left(\alpha ,r\right)=g_{2}\left(\alpha ,r\right)\left[g_{2}\left(\alpha ,r\right)+2g_{4}\left(\alpha ,r\right)\right]$$

$$f_{3}\left(\alpha ,r\right)=g_{3}\left(\alpha ,r\right)\left[g_{3}\left(\alpha ,r\right)+2g_{4}\left(\alpha ,r\right)\right]$$

$$f_{4}\left(\alpha ,r\right)=g_{4}{\left(\alpha ,r\right)}^{2}$$

where *g_i_*(*α*,*r*) (*i *= 1, 2, 3, 4) are as given below:

$$g_{1}\left(\alpha ,r\right)=\left(2-\alpha \right){\left(1-r\right)}^{2}/4+r\left(1-r\right)/2+\left(1-\alpha \right)r^{2}/6$$

$$\begin{array}{c} g_{2}\left(\alpha ,r\right)=\left(1-\alpha \right)r\left(1-r\right)/3+\left(10-\alpha \right)r^{2}/36\\ g_{3}\left(\alpha ,r\right)=r\left(1-r\right)/2+\left(8+\alpha \right)r^{2}/36\\ g_{4}\left(\alpha ,r\right)=\left(2+\alpha \right){\left(1-r\right)}^{2}/4+\left(2+\alpha \right)r\left(1-r\right)/3+\left(2+\alpha \right)r^{2}/6 \end{array}$$

The maximum likelihood estimate (MLE) of the recombination frequency in diploids and allotetraploids can be calculated from solving the normal equations:

$$\partial L\left(r|n_{1},n_{2},n_{3},n_{4}\right)/\partial r=0$$

which is a quadratic equation of *r*. In the diploid population where *n*_1_= 2805, *n*_2_= 322, *n*_3_= 333 and *n*_4_= 791, the quadratic equation has only one real root filling in [0.0, 0.5], which is the MLE $\hat{r}=0.1643$. Based on the likelihood function, one can calculate the standard deviation of the estimate from the Fisher's information measure for MLE. In the present context, it equals:

$${\sqrt{-1/\left[\partial^{2}L\left(r|n_{1},n_{2},n_{3},n_{4}\right)/\partial r^{2}\right]}}_{r=\hat{r}}=0.0062\;\left(\text{Table 1}\right).$$

In their original report [4], Pecinka *et al. *estimated the recombination frequency by equating the probability of the recombinant individuals, in other words, those displaying green and red seeds, to the observed proportion of these individuals. In the present notations, the probability has a form of 2*r*-*r*^2 ^= 2(*n*_2_+*n*_3_)/*n *where *n *= *n*_1_+*n*_2_+*n*_3_+*n*_4_. They provided an estimate of $\hat{r}=0.154$ with a standard deviation of 0.009. It is not clear how the standard deviation was calculated. The method for calculating the recombination frequency may not be statistically appropriate in two aspects. Firstly, the calculation did not use the full information of the data. For example, the individuals with yellow seeds were not taken into consideration when counting for recombination events. In fact, there is a proportion of [*r*(1-*r*)+*r*^2^/2]/[3(1-*r*)^2^/4+*r*(1-*r*)+*r*^2^/2] among this group of individuals which carry recombinant gametes. Secondly, the MLE of *r *obtained from the present analysis is four times as likely as that provided by the original report, that is:

exp[L(r=0.1643)-L(r=0.154)]=4.096.

In the allotetraploid population where *n*_1_= 1484, *n*_2_= 275, *n*_3_= 298 and *n*_4_= 320, the MLE and corresponding standard deviation are calculated as 0.2770 ± 0.0110. The estimate is in contrast to 0.241 with standard deviation = 0.018 in the original report.

To analyze the likelihood model for the autotetraploid marker data, we firstly noticed that the parameter α involves information of allele segregation at the green marker only. By setting *r *= 0 in the phenotypic probabilities given above, we can work out:

$$p_{g}\left(\alpha \right)=f_{1}\left(\alpha ,0\right)+f_{2}\left(\alpha ,0\right)=\left(12-4\alpha -\alpha^{2}\right)/16$$

$$p_{0}\left(\alpha \right)=f_{3}\left(\alpha ,0\right)+f_{4}\left(\alpha ,0\right)={\left(2+\alpha \right)}^{2}/16$$

which represent the probability of observing an individual carrying or not carrying the green fluorescent marker respectively. Let *n_g _*= *n*_1_+*n*_2 _be the number of individuals carrying the green marker allele and *n*_0 _= *n*_3_+*n*_4 _be the number of individuals not carrying the allele. The log-likelihood function has the form:

$$L\left(\alpha |n_{g},n_{0}\right)=n_{g}Log\left[p_{g}\left(\alpha \right)\right]+n_{0}Log\left[p_{0}\left(\alpha \right)\right]$$

Solving the following equation:

$$\partial L\left(\alpha |n_{g},n_{0}\right)/\partial \alpha {|}_{n_{g}=14575,n_{0}=5314}=0$$

results in the MLE of $\hat{\alpha }=0.0676$ with a standard deviation of 0.0121 (Table 1).

Incorporating $\hat{\alpha }=0.0676$ into the likelihood function, we found that the equation:

$$\partial L\left(\hat{\alpha },r\right)/\partial r{|}_{n_{1}=12707,n_{2}=1868,n_{3}=2216,n_{4}=3098}=0$$

has only one real root, $\hat{r}=0.3048$, which is the MLE of the recombination frequency under the tetrasomic model. The estimate has a standard deviation of 0.0051, which was calculated from the second derivative of the likelihood function at the MLE. Based on the estimates of α and *r*, we can predict the coefficient of double reduction at the red marker from: $\hat{\beta }=\left[\hat{\alpha }{\left(3-4\hat{r}\right)}^{2}+2\hat{r}\left(3-2\hat{r}\right)\right]/9=0.1857\;\left[12\right]$

which is below the upper bound of the maximum value of double reduction ¼ [12]. The estimates of α and β strongly suggest multivalent pairing among the homologous chromosomes and, in turn, double reduction occurring in the genome region flanked by the fluorescent markers (Table 1).

## Discussion

This article presents likelihood-based methods for estimating the recombination frequency and other relevant parameters from segregating populations of diploids, allotetraploids and autotetraploids. We demonstrate the methods by re-analyzing the datasets published in Pecinka *et al. *[4]. Re-analysis of the datasets with the methods developed here reveals quantitative and qualitative differences from the original analysis. Firstly, unlike Pecinka *et al.*, the present analysis enables estimation of double reduction at the marker loci under study and discovers significant double reduction at the markers, emphasizing the necessity of taking the tetrasomic nature into consideration in the data analysis. Secondly, the present study differs from the original analysis in the inferred order in frequency of meiotic recombination of allotetraploid and autotetraploid *Arabidopsis*. Pecinka *et al. *concluded that meiotic recombination was more frequent in the allotetraploids than in the autotetraploids whilst our results predict differently. Rationale for the present prediction is supported by several aspects. In fact, allotetraploids show strictly disomic inheritance particularly in the present instance where homoeologous chromosome pairing was excluded. In contrast, homologous chromosomes in autotetraploids have a substantially higher chance to pair, which is essential for recombination to occur among the chromosomes [13]. In addition, it has been theoretically demonstrated that autotetraploids have a much higher upper bound value of recombination frequency in autotetraploids (¾) than that in diploids and allotetraploids (1/2) [14]. Thirdly, the estimates obtained from the present analysis are significantly highly supported by the observed experimental data when compared with those from the original analysis in term of likelihood values of these estimates.

## Computer program

Formulation and numerical analysis represented above were programmed in Mathematica [15] and the programs will be made available from the corresponding author after publication of this paper.

## Competing interests

The authors declare that they have no competing interests.

## Authors' contributions

ZWL conceived the research and formulated the statistical analysis. LW and ZWL analyzed the data and wrote the paper. Both authors read and approved the final manuscript.
